# Improving access, mixed continuity: effects of multidisciplinary teams on primary health-care in Finland – a quasi-experimental study

**DOI:** 10.1080/02813432.2025.2502658

**Published:** 2025-05-08

**Authors:** Elisa Jokelin, Laura Piirainen, Erja Mustonen, Paulus Torkki

**Affiliations:** a Department of Public Health, University of Helsinki; b Western Uusimaa Wellbeing Services County and Department of General Practice and Primary Health Care, University of Helsinki; c Ministry of Social Affairs and Health

**Keywords:** The quadruple aim (QA), chronic care model (CCM), Open access (OA), Lean management, continuity of care (COC)

## Abstract

**Objective:**

The multidisciplinary team (MDT) approach in primary care is a relatively recent innovation, developed over the past 15 years. There is limited data on MDTs’ effects on Quadruple Aim (QA) goals. The object of this study is to evaluate the implementation of a novel MDT from 2021 to 2023 and its impact on access and continuity of care, compared to an established model. Future research will explore its effects on staff satisfaction, costs, and health outcomes.

**Design, setting and patients:**

This quasi-experimental study compares five intervention health centers with three control centers. It includes all primary care patients from 2021 to 2023, presenting data on access and continuity before and after the intervention.

**Intervention:**

Nurse-only consultations were replaced with a multidisciplinary nurse-physician model to address issues during initial contact more effectively. Nurses also took on the role of case managers, enhancing relational continuity. Lean daily visual management with continuous improvement, strategic goal setting, and coaching leadership style were implemented.

**Main outcome measures:**

Access was measured using the ‘third available appointment’ (T3) metric, and continuity with the COC-index, both for physicians only.

**Results:**

Access improved at all intervention centers, with T3 reduced from 90 to 1.125–4.75 days, while controls remained at 90 days. COC improved at three intervention centers but declined at two, with declines also observed at control centers.

**Conclusion:**

The novel MDT enhanced primary care access compared to the traditional model. However, relying solely on T3 may be insufficient for evaluating effectiveness. Mixed results in continuity underscore the need for further investigation.

## Introduction

Challenges within the current health services include diminishing tax revenue, the aging population, and increasing demands on the quality of medical services and medical consultations and treatments. These pressures have motivated a demand for structural improvement across Scandinavian health care systems [1–4]. Finnish reforms called for multidisciplinary team (MDT)-based delivery of primary health care services; this came despite limited data and scientific back-up. The ‘Future Social and Health Centre’ program (launched in 2020) aimed to improve equal access, timeliness, and continuity of services, shift the focus of activities towards preventive and proactive efforts, ensure the quality and effectiveness of services, and strengthen the multidisciplinary and interoperable nature of services [1]. This aligns with international trends towards establishing integrated or team-based care in responding to the increasing global demand on health care and the additional burden of chronic diseases [5]. In our study, a multidisciplinary team refers to the integration of all healthcare professionals at the health center into immediate communication, working together within standardized processes, managing work in shared manner and working together on continuous improvement to increase fluency. The objective is that when a patient initiates contact, they can collectively assess which professional is best suited to assist them, and the case is then directed to that professional, e.g. in the case of typical back pain to a physiotherapist. This approach supports the vision of the Future Social and Health Center program to provide ‘services with a single contact’ [1].

Access to health care was defined in 2013 framework as a multi-dimensional view incorporating health system’s attributes of approachability, acceptability, availability, affordability, and appropriateness, with socio-economic determinants by the individuals to perceive, to seek, to reach, to pay, and to engage in health care [6]. In our study, we consider access how it describes the possibility to reach (Levesque’s approachability and ability to seek and reach), and access health care (availability and ability to engage), and to get the service you need (availability, appropriateness, and ability to engage).

Continuity of care is considered in our study as interpersonal continuity as described by Saultz and Haggerty et al. [7,8]. The choice to measure and focus on interpersonal continuity is for its strong association with positive health outcomes [9].

Few studies have reported the impacts of team-based care on access or continuity. In 2015, a family health teams’ association in Ontario Canada, documented that patients served by team-based services reported better access to primary care than Ontario residents in general [10]. Contrarily, a 2017 Canadian study found no association between team-based care and access [11]. In Germany, a 2017 report on innovation in team-based care documented enhanced access to primary care services, although this intervention included several components beyond a multidisciplinary approach [12]. Similarly, a 2018 systematic review in the UK documented that integrated care might enhance access to services [13]. In another 2018 Canadian study, 51.5% of patients reported improved access to care with a team-based approach [14]. A 2019 qualitative study on academic clinics found that team-based organization provided better same-day access and continuity for chronic care patients [15]. Finally, a 2020 study in the USA redesigned team roles within a general practitioner practice, resulting in better access by increasing visits per day without adding extra personnel [16].

Existing literature generally lacks proper descriptions of the MDT interventions and comprehensive assessments of the impacts on several relevant aims. More precisely, there is a shortage of evidence that clearly indicates whether team-based care produces good access to services while maintaining continuity of care, and which combines it with other Quadruple Aim (QA) goals. This report aims to address this knowledge gap. We describe the logic of the MDT-based care innovation, the composition of the MDT, and its roles, workflows, management, and supporting contextual frameworks.

### The aim of this study

Objective of this study is to present the results of implementing a novel MDT-model for primary care in Finland, focusing specifically on its impact on access to care and continuity of care. The MDT model was studied using a quasi-experimental design in health centers in the City of Espoo. While this report centers on access and continuity, effects on other aspects such as care quality, costs, and staff experience will be addressed in future work as a part of a wider study.

## Materials and methods

### Intervention design

**Figure 1.**
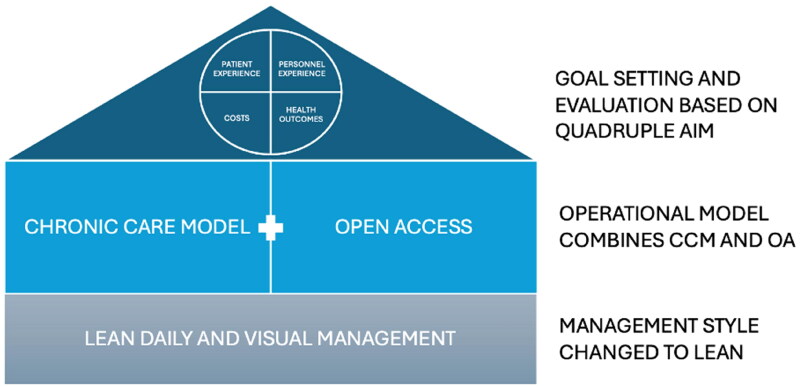
The logic of the MDT intervention elements.

We developed the MDT utilizing the best practices and latest knowledge. Figure 1 describes the logic of the MDT intervention elements and how they are related to each other. First, QA was selected for performance measurement and evaluation of the team model as it is one of the most used frameworks nowadays [17]. Second, the Chronic Care Model (CCM) and Advanced or Open Access (OA) have been identified as effective operational principles in health center practices [18,19]. In services, lean visual daily management and continuous improvement cycles have been found to play a significant role in enhancing performance [20,21]. Consequently, we adopted Lean management as our management system, given its proven effectiveness across various sectors.

#### Chronic care model

CCM describes the elements needed for a robust health care system to provide high-quality care to people with chronic conditions. The model comprises six elements: (1) an organization and leadership that commits to providing safe, high-quality care, (2) community resources that have been mobilized to meet patients’ needs, (3) patients empowered in self-management of their health issues, (4) system designed to provide proactive care that employs the talents of all team members and gives case management, (5) built-in decision support, and (6) the clinical information systems to support all this [18]. Although the model was built over 30 years ago, the CCM still provides accurate guidelines for a system to provide care for people with chronic conditions. Of these guidelines, our intervention incorporates multidisciplinary team design by adding multidisciplinary consultation opportunities to the team processes and daily management.

#### Advanced access or open access

Access to primary care has been a challenge in Finland for years [22]. Although the situation has improved (in October 2019 40% of patients got an appointment with a doctor within a week and in March 2024 this had risen to 60%) the speed of access is still far from ideal [23]. Traditionally, the workflow is as follows: when contacting a Finnish health center, a call is usually registered in a call-back system and a nurse will call back to the patient. Waiting time may extend to several days, for non-urgent needs [24]. A nurse evaluates whether health care is needed, and which professional would best attend to the person’s needs. If necessary, the nurse consults a physician about the patient’s issue; the physician would then opt to confer with the nurse, directly call the patient, or invite the patient for an on-site reception at the health center. Recently, video consultations have also become available.

In the classical Advanced or OA model, if a patient has a health-related issue, the healthcare provider would attempt to schedule an appointment for the same day [19]. Our MDT takes this a step further. Under our approach, the professional starts to solve the patient’s issue upon first contact, regardless of whether the initial contact was achieved *via* phone call, online messaging, or walk-in consult. This obviates any need for triage: we can directly focus on the patient issue. Acceptable reasons for postponing the resolution include patient’s wishes, if the issue cannot be resolved at the time, or to preserve continuity of care (for example, if the patient’s personal physician is not currently on-shift).

Our model design was informed by earlier studies which focused on reducing waiting times. A 2017 review [20] identified 11 studies implementing interventions to reduce waiting times. Every intervention in this review incorporated OA scheduling in one way or another. In OA (or Advanced Access) scheduling, part of each workday is usually reserved for same-day patients, without assessing the urgency of the need [19]. Other major elements which improved access were redirection of workload from physician, reduction of follow-up efforts, increased use of telephone, nurse and physician triage, incorporation of nurse practitioners, promotion of selfcare, and use of email. Of these methods, redirection of workload from physician, the increasing use of telephone, physician triage and promoting selfcare are integrated into the model.

#### Lean management

Lean thinking has come to health care as one of the main ways to improve value for patients with a comprehensive approach. All key principles of successful lean implementation have been integrated into our MDT: (1) continuous improvement sessions are a part of the teams’ daily and weekly routine, (2) the teams were coached on value on the patient’s perspective, (3) goals in line with the organizational strategy were set on team and health center level, (4) managers were coached to implement coaching leadership, (5) daily visual management boards and routines were implemented, and (6) main standard processes were built and implemented.

### Description of intervention

**Figure 2.**
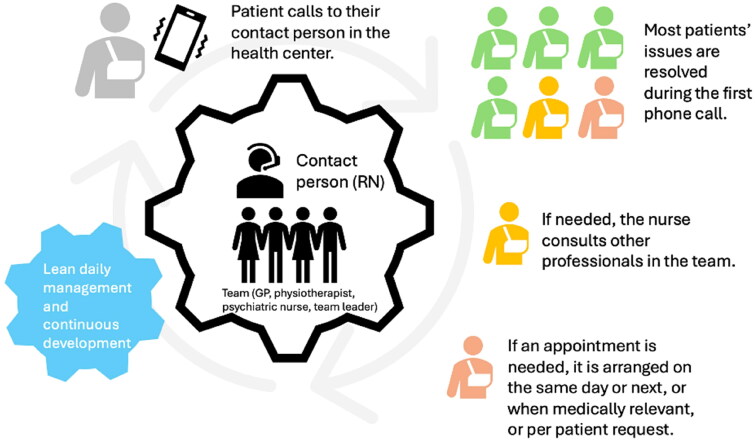
A visualization of the patient’s journey within the team model. Adapted from visualization by Maarika Autio (RN = registered nurse, GP = general practitioner).

During the intervention, the focus of the operation shifted from managing the queues and the number of reception timeslots towards managing the patient’s case (Appendix 1, supplementary material). Under the new operating paradigm, i.e. after the intervention, new emphasis was placed on avoiding queuing and pushing to achieve the most progress possible upon the initial contact (Figure 2). The most suitable professional was recruited to answer the patient’s need/concern during the initial on-site or remote reception. The MDT approach alters the selection process for physician receptions but does not specifically account for the chronicity of the patient’s condition. Previously, during the initial contact, the nurse determined whether a patient needed to see a physician and how urgently. In the MDT however, the nurse consults with a physician to jointly decide on the appropriate next steps and scheduling. As a result, every patient requiring a physician’s appointment receives one. For patients whose issues can be resolved immediately over the phone, this is done in consultation with the physician. Alternatively, if the consulting physician judges that the patient would benefit from continuity, the case can be referred to a physician more familiar with the patient’s history. The patient was asked to attend the reception in person if this was required for medical treatment. Proactive monitoring was emphasized in remote care. During the initial consult, the ability to remotely decide between remote vs. on-site care is influenced by factors including patient preference, the medical issue, and the professional’s experience. Over time, we observed a clear increase in preference for remote care, as well as an increase in the number of consultations between medical team members. This aligned with the stated purpose of attempting to finalize care of all patients during the same day, without any urgency triage. When it became impossible to meet the entire demand on any day, root-cause analysis and other problem-solving efforts led to the identification of possible corrective measures. Additionally, the nurse *via* whom the patient makes initial contact with the MDT unit would automatically be assigned responsibility for any subsequent case-handling and/or follow-up. Subsequent patient contact to the MDT would automatically be routed directly to the nurse’s telephone line. Nurses would then consult the supervising physician in the team room or leave a message for the physician that had previously handled this patient’s case. In the case of chronic disease monitoring, the nurse acts as a personal care coordinator in charge and consults the physician when necessary, e.g. the results do not reach the goals set by the physician. In a situation of limited resources, this focuses the physician’s work on treating specially those patients who need a physician’s assessment either in acute or chronic disease, in other words, leads to more reasonable utilization of the physician’s work and reduces physician’s pressure and need for reception times.

During the intervention, we noticed clear trends towards daily deviation tracking, continuous improvement, and systematic problem solving. The health-center management teams increasingly began to favor visual day-to-day monitoring of process indicators for the MDTs, and management of the health center *via* the monitoring of team and center key-performance indicators.

In summary, our MDT combines CCM, OA and lean management in the following way: when a patient contacts the MDT, their case will be resolved immediately (as per OA and Lean methodologies), in a multidisciplinary way wherever possible (CCM), and if they need an appointment, one will be scheduled without an urgency assessment (OA). While managing the case, the patient is proactively supported, with emphasis placed on self-care (CCM). Activities are guided by visual day-to-day management and strategic indicators, and problems are solved systematically as part of the team’s daily routine (Lean).

### Implementation of intervention

**Figure 3.**
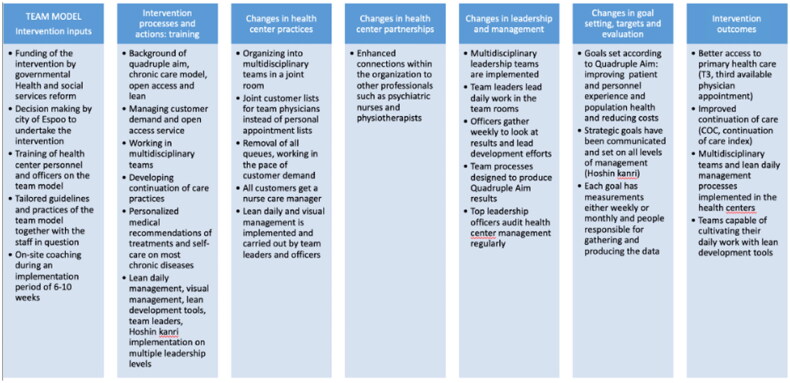
Logic model for the MDT intervention, modified from Moore et al. 2015 [25].

The intervention was implemented in the city of Espoo (pop. 314 150), with participating health centers selected by the chief executive medical officers. Figure 3 presents details of the logic implemented by the intervention. Consultation company Medielli Oy was selected to help implement the intervention model.

An extensive 6–8-week coaching program within the health centers allowed officers, team leaders, physicians, and nurses to become familiarized with the operating workflows and principles of the intervention. During approximately 130 h of specialized leadership sessions, officers were coached on how to establish team vision and goals (that align with organizational strategy), set targets, evaluate the current state of leadership and development, assess demand, implement lean methods in daily management and workflow re-design, and lead change. Team leaders were selected and then coached (for approximately 30 h) on daily management, lean problem solving and facilitation methods. Physicians and nurses were provided access to 25 h of coaching workshops, to re-design operating workflows and processes to implement the new strategy and achieve QA goals. The backlogs were handled, and patient lists were empty prior to implementation of the intervention.

**Figure 4.**
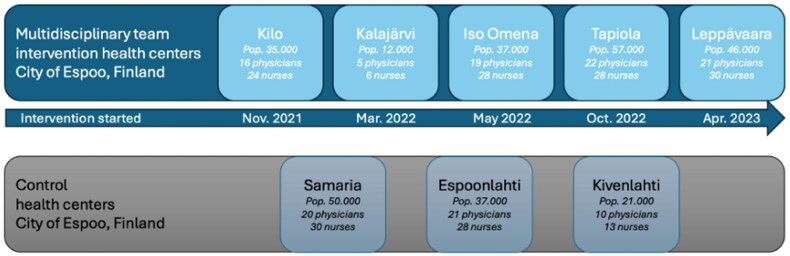
Intervention and control health centers.

The roll-out of the intervention in the five intervention health centers lasted 18 months altogether (Figure 4). Intervention elements were further refined during this time, to emphasize improved continuity of care (COC) and personnel well-being. Control health centers continued with their usual model of care, except for Samaria which was designated an ‘Infection Center’ between March 2020 and August 2021 during the acute phase of COVID-19 epidemic. In Finnish primary healthcare, each health center is responsible for its own population, and variations in patient background characteristics occur randomly. For this reason, we have not considered it meaningful to describe the sociodemographic background factors of patients, as there is no evidence to suggest significant variation between health centers, nor does the intervention influence patient selection.

### Data collection

Maturity levels of the intervention elements were assessed *via* a questionnaire in February 2024 (Appendix 2, supplementary material). The chief officers in each health center were provided five questions with which to assess their situation on a scale of 1–5 (1= least-, 5 = most-mature): (1) multi-professionalism and roles in triage, (2) continuity of care, (3) continuous development, (4) management, and (5) supply and demand. The full questionnaire) is in Appendix 2 (supplementary material). The questionnaire was also shared with the control centers’ officers, who assessed development changes in control centers’ management and processes. The questionnaire was developed by the coaching team based on their experience on the MDT elements and goals.

For physicians, third available appointment (T3) and COC-index metrics were used to assess access to services and continuity of care [20,26]. T3 was calculated at each health center and documented weekly by its executive officers. The Continuity of Care -index (COCI) is calculated by evaluating the distribution of a patient’s contacts (on-site visit, phone, or video consultations) among physicians [27]. The index ranges from 0 to 1, with values close to 0 indicating low continuity, characterized by visits distributed among multiple physicians, while a value of 1 represents complete continuity, where all visits are conducted with the same physician. COC index was retrospectively extracted from the ‘LifeCare’ electronic health record monthly. The COC assessment period is a rolling year consisting of the 12 full calendar months preceding the day selected. The data used for the COC calculation was filtered to exclude patients who made fewer than three visits to physicians at a given center during the 12-month COC assessment window. The months included in the pre-post analysis varied depending on when the intervention was implemented at each health center. Data were collected over a three-year period, from January 2021 to December 2023. Table 1 presents the durations of the observation periods before and after the interventions. For the control units, follow-up data were collected for time periods corresponding to those of the intervention units.

**Table 1.** The intervention month, pre-post analysis periods and months, and number of patients in the COC calculation for each health center.

Health center	Intervention month (not included in the analysis)	Pre-intervention period for T3	Pre-intervention period for T3 (mo)	Pre-intervention period for COC	Pre-intervention period for COC (mo)	Post-intervention period for T3	Post-intervention period for T3 (mo)	Post-intervention period for COC	Post-intervention period for COC (mo)	Mean number of patients per month in the COC calculation (SD)
Kilo	Nov.21	Jan.21 to Oct.21	10	Aug.21 to Oct.21	3	Dec.21 to Dec.23	26	Oct.23 to Dec.23	3	1959 (432)
Kalajärvi	Mar.22	Jan.21 to Feb.22	14	Dec.21 to Feb.22		Apr.22 to Dec.23	21			682 (162)
Iso Omena	May.22	Jan.21 to Apr.22	16	Feb.22 to Apr.22		Jun.22 to Dec.23	19			1441 (139)
Tapiola	Oct.22	Jan.21 to Sep.22	21	Jul.22 to Sep.22		Nov.22 to Dec.23	14			2509 (526)
Leppävaara	Apr.23	Jan21 to Mar.23	27	Jan.23 to mar.23		May.23 to Dec.23	9			2988 (249)

### Statistical methods

The design is quasi-experimental, creating a benchmark-controlled study [28]. The effects on COC and access to services were assessed comparing five intervention health centers (1) before and after the intervention and (2) after the intervention with the three control health centers [29]. First, the normality of the distribution was assessed with Shapiro-Wilk -test and statistical analyses were made with t-test (normal distribution) or with Mann-Whitney u-test (non-normal distribution).

### Ethics

Several ethical considerations were addressed. Patient preferences regarding care continuity were documented and respected. The intervention was designed to minimize harm by avoiding abrupt transitions between providers and maximizing benefits through coordinated care plans. Non-discriminatory access was ensured, with equitable resource distribution to address disparities in care access. Patient data was handled securely across teams. Clear roles and accountability were established to prevent care fragmentation, with systems to track unresolved referrals. The intervention was clearly differentiated from usual care practices, with adherence to reporting standards for complex interventions. This study was approved by the ethical committee of University of Helsinki (January 2022).

## Results

**Figure 5.**
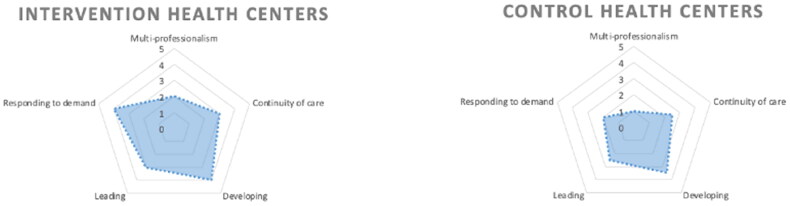
Maturity levels of the multidisciplinary team model elements in intervention and control health centers (*N* = 5 for intervention health centers and *N* = 3 for control health centers).

Differences observed in maturity levels of the intervention elements are presented in Figure 5. The greatest differences between intervention and control health centers were for ‘Responding to demand’ and ‘Multi-professionalism’.

### Impact of the intervention on access to care

**Figure 6.**
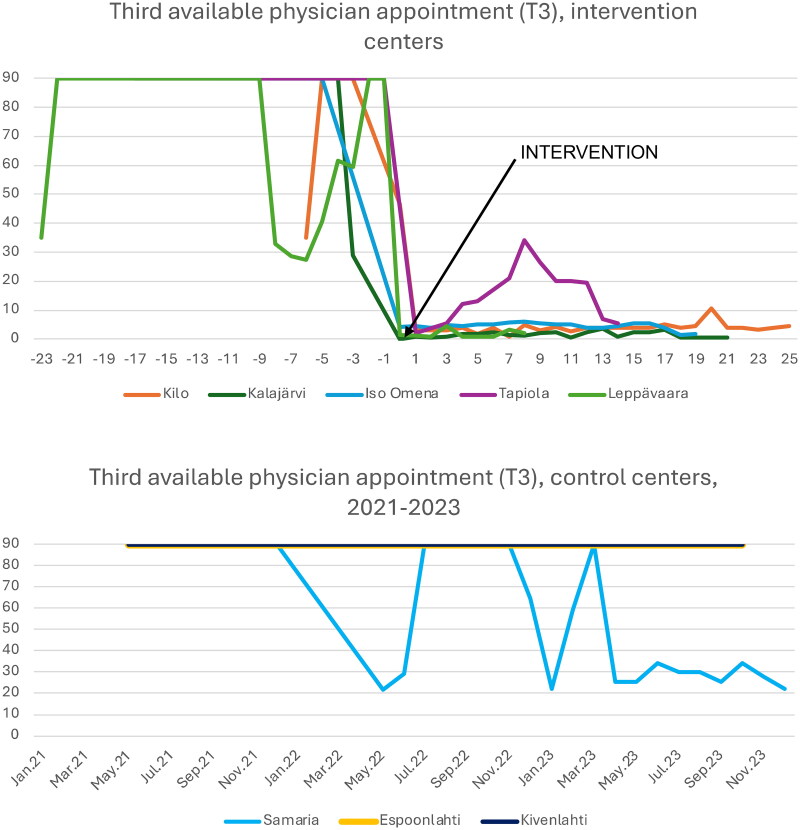
Third available physician appointment (T3) values observed at intervention and control centers. For intervention health centers, the horizontal timeline expresses the number of months pre- (negative numbers) and post- (positive) intervention (which occurs at zero). Observed T3 data for control health centers is presented for the interval between January 2021 and December 2023.

At all intervention centers, the access to care measured by an average T3 metric decreased from 90 days (pre-intervention) to 1–5 days post-intervention (*p* < 0.001) (Table 2, Figure 6). Difference-in-differences shows reduction of 81–99% in waiting times in the intervention centers while control centers’ waiting time median did not decrease (*p* < 0.001). In the context of these health centers, ‘T3 = 90 days’ indicates a theoretical upper limit. Without MDT intervention, therefore, if the waitlist was already long enough and a patient’s issue was deemed non-urgent, that patient could be told that ‘no appointment times are available’ and then encouraged to call back ‘at a later date’. Although a mix of higher/lower T3 values were also observed post-intervention, we note that no intervention center presented T3 values at or above 15 for the time periods more than 13 weeks post-intervention. This contrasts clearly with observations at the control centers, since no control center in this study presented T3 values less than 20 during the entire period of the assessment. This suggests an apparent reduction of the worst-case T3 allowed by the intervention, although future efforts would best validate this initial observation.

**Table 2.** Third available physician appointment and continuity of care, comparisons, and statistical significances (bold if p less than 0,05).

Third available physician appointment			Before and after intervention		Intervention center (after) compared to controls (after)
Health center	Before intervention (median)	After intervention (median)	*P-value*	After intervention, controls	*P-value*
Kilo	90	4	**< 0.001**	90	**< 0.001**
Kalajärvi	90	1,5	**< 0.001**	90	**< 0.001**
Iso Omena	90	4,8	**< 0.001**	90	**< 0.001**
Tapiola	90	17	**< 0.001**	90	**< 0.001**
Leppävaara	90	1,1	**< 0.001**	90	**< 0.001**
Continuity of care			Before and after intervention		Intervention center last three months’ mean compared to controls last three months’ mean
Health center	Three months before the intervention, mean	Last three months of follow up, mean	*P-value*	Last three months of follow up, control centers, mean	*P-value*
Kilo	0.536	0.377	**< 0.001**	0.218	**< 0.001**
Kalajärvi	0.446	0.335	**< 0.001**		**< 0.001**
Iso Omena	0.183	0.225	**0.002**		**0.089**
Tapiola	0.227	0.314	**< 0.001**		**< 0.001**
Leppävaara	0.214	0.291	**< 0.001**		**< 0.001**

### Impact of the intervention on continuity of care to a physician

**Figure 7.**
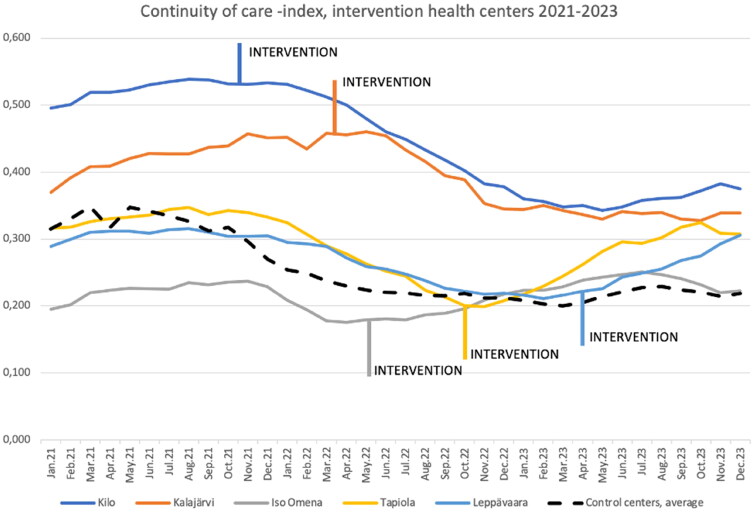
Physician continuity of care in intervention health centers (colored lines) and control centers (dashed line, average).

We observed a decrease in physicians’ COC at two intervention health centers, but physicians’ COC increased at three other intervention centers after the intervention (Table 2, Figure 7).

## Discussion

This study presents observations made during the application of MDT to deliver primary care in a challenging and dynamic environment; we observed simultaneous improvement in access while continuity of care presented mixed results. Clear post-intervention improvement to access to care was observed at intervention centers (relative to control centers). Although a mix of higher/lower T3 values were also observed, we note that no intervention center presented T3 values at or above 15 after more than 13 weeks post-intervention, whereas no control center presented T3 values less than 20 during the entire period of the assessment. This suggests an apparent reduction of the worst-case T3 allowed by the intervention. Future efforts may yet validate this initial observation.

Based on these data, this MDT intervention contains elements that apparently served to increase access (i.e. open access scheduling, multi-professional consultation, physician triage, lean management). This was also visible in the maturity levels of the intervention elements (responding to demand, multi-professionalism). Post-intervention access to services at the intervention health centers was consistently observed at an improved level; this also continued to hold true after the end of the follow-up period. The MDT approach has also been maintained as the operational model at the intervention centers.

Focus on access to care can be contested when it is often contradictory to continuity of care. The national discussion served as a significant driver in combining access and continuity as concurrent goals. In response to persistent challenges in accessing primary care, the Finnish government enacted legislation in September 2023 that reduced the maximum waiting time for non-urgent care from three months to two weeks [24]. This mandates that patients must be seen by a physician within 14 days once a need for medical attention has been determined. This led to increased efforts throughout the health care system to reduce waiting times. Access to healthcare took another step backward when the government reinstated a maximum three-month waiting period for treatment at the beginning of 2025 [30].

A more comprehensive and patient-centric approach to access would include other measures in addition to T3 [6]. Third available appointment encompasses merely the possibility to use healthcare resources in a timely manner, whereas other dimensions e.g. to identify, to seek, to reach, and to be offered, may be more relevant to many patients’ needs. For instance, a potential risk arises in multiprofessional consultation practices. Patients may perceive a telephone consultation with a nurse as being of lower quality compared to an on-site appointment with a physician, even if the nurse relays the physician’s advice and provides an immediate response. This perception may persist despite the convenience of avoiding a prolonged wait for an on-site reception with the physician. We were able to cut down the waiting times and pressure for physician’s on-site appointments as we re-evaluated which patients truly need physician’s assessment and who can be helped otherwise, for example, nurses took responsibility of chronic disease monitoring in controlled manner if the results reached the goals set by the physician. In this way, we were able to confirm the physician’s effort in diagnosing long-term diseases and creating patient-centered treatment plans. In the multidisciplinary team (MDT) model, physicians allocate a significant portion of their work time to nurse consultations. Despite efforts to optimize workflow using Lean management principles, there was a noticeable decline in the number of physicians’ on-site appointments. This decrease was not uniform, varying across different health centers and fluctuating from month to month. Additionally, a similar reduction in on-site physician appointments was observed in the control health centers over time (L.Piirainen, personal communication 19.11.2024).

We note three main insights about the continuity of care. First, the prior model produced better continuity of care at two intervention centers. Both health centers have a history of permanent physician personnel with long and stable patient-physician relationships. It is up to the decision-makers to balance these aspects since these health centers’ history shows that excellent continuity could not co-produce good access. Within the study, the intervention elements were intensively developed to better consider continuity of care as soon as this evolution was noted. Second, three other intervention centers benefited from the experience in the first two (L.Piirainen, personal communication 19.11.2024) and were able to recover better than controls from the devastating impact on continuity of care of COVID-19 pandemic while the COCI remined low in control centers (L.Piirainen, personal communication 19.11.2024). Thirdly, the team model in question seems to produce physicians’ COC-index of approximately 0.3, so if previously it was higher, it tends to decrease, and if lower, it increases. Speculation for a reason for this could be related to the size of the team in question. Teams in this study have an average of 12–18 members of which 4–6 are physicians. Although the teams are trying to manage the continuity, the MDT model should be further developed to increase the COC from 0.3 which the authors do not consider sufficient.

All health centers included in this assessment utilize physicians in residency, so part of the workforce is inherently non-permanent. Combining the education of new clinicians and provision of high levels of continuity can pose a challenge; this is not insurmountable [15]. Finnish medical schools require that all physicians, regardless of specialty, spend 6–9 months delivering primary care during their training period. This provides a steady supply of young physicians to health centers but also restricts the maximum possible continuity of care that can be achieved at a given center. Some health centers compensate by restricting the assignment of chronic-disease cases exclusively to their permanent personnel, but this makes it difficult for young physicians to learn about this crucial field within the primary care setting.

Previously, only few studies have combined reporting on COC and access to care. In a 2018 study of an intensive program for high-needs patients confirmed its ability to simultaneously increase continuity and access [31]. A 2020 study used lean development cycles to increase the patients’ awareness of their own care team’s after-hour services; this resulted in the decreased use of outside service providers [32]. In 2020 a retrospective cohort study investigated physicians in a primary care network and their access and continuity of care; the authors reported that diminished access led to decrease in continuity and increased emergency department use [33]. However, a 2022 study presented data supporting the reverse association; explicitly naming the general practitioner increases self-reported access to care [34]. A recent article calculating the effects of different operational models to continuity of care suggests that the team composition and number of professionals has a mathematical correlation on how the continuity of care develops [35]. No studies have yet been found which report on utilizing an MDT approach to simultaneously increase access and continuity. We believe this encourages additional research in this area.

Our study has the novelty of simultaneously utilizing team-based care in pursuing goals, describing the intervention with detail and the underlying frameworks, and reporting its impact on access and continuity. Studies on team-based care, access, and/or continuity, either lack a detailed description of the model [10,11], don’t report access [12] or continuity [13,14], or describe only qualitative impacts [15]. This study also adds knowledge on what are MDT’s effects on Quadruple Aim goals. It seems a promising operational model to be used in a situation where lack of personnel, especially of primary care doctors, is challenging the quality of care. Based on our findings it is reasonable to say that the MDT increased access which could be seen most directly to enhance patient experience but also contributing to improving population health by the increased access [36,37].

It is not clear whether interpersonal continuity, which COC-index measures, is always superior to team-based continuity, especially with patients with chronic diseases. Team-based continuity was not assessed in this study but previous research has shown it to have good impacts on health of chronic disease patients [38–40]. The relevance of type of continuity of care to the patient can vary depending on the context [8]. In a team-based care model, relational continuity may take a secondary role, influenced by the number of physicians involved and the specific practices implemented to maintain continuity. However, from the perspectives of informational and management continuity, the team-based model is often more effective. This is because a smaller, cohesive team can better manage and retain a comprehensive understanding of the patient’s condition. In contrast, in traditional models—particularly in Finland, where maintaining relational continuity is often challenging—continuity is primarily achieved through information systems. Unfortunately, this approach can obscure the overall picture of the patient’s situation, making it difficult for healthcare providers to fully grasp and address their needs. Relational continuity was chosen as the type of continuity to be assessed because of its strong association with relevant patient outcomes [9]. According to our experience, even though a larger team size challenges continuity of care, it is often preferred by health center officers as it facilitates managing absences and other daily management challenges. Therefore, it is important to incorporate components that support the continuity of the patient-physician relationship into the MDT model.

### Changes and confounding factors in the context during the intervention period

This evaluation of the health centers of the Espoo city was conducted from January 2021 to December 2023. However, underlying factors affecting the continuity and access to care, already exists earlier, particularly at the year 2020. These surrounding factors lead to reduction in continuity of care in all health centers, independent from the intervention.

The COVID-19 pandemic changed the Finnish health care system in March 2020. Because of the pandemic, the assessment of the need for care was changed from face-to-face visit with a nurse to phone lines. After first decrease in demand after COVID-19 started, the quantity of patient contacts dramatically increased in a short time. This led to increased queuing and greater burden on the health service overall, which in turn resulted in a reduction in access and lower availability of care. The constantly changing COVID-19 guidelines and the load caused by infection tracking exhausted health care professionals at health centers. The availability of services deteriorated, leading to the accumulation of unmet medical needs and continued poor availability. In Espoo, the control health center Samaria became an infection center between March 2020 and August 2021, and mainly tasked with responsibility for primary care provision to COVID-infected patients during this time. Meanwhile, other health centers were assigned responsibility to care for the long-term illnesses of patients living in the Samaria region. At the Samaria region, the treatment of long-term illnesses was deprioritized, which led to significant decline in continuity of care.

Consequently, at the beginning of the intervention, continuity and access to care were effectively reduced, while its healthcare professionals’ job satisfaction was also lowered. We noted, too, that staff availability was significantly reduced at some of the health centers. The need to find a new approach to patient care was identified in Espoo. In our first intervention health center, Kilo, the professionals were exceptionally committed and persistent, which is evidenced by particularly good continuity of care in the initial period of the study. Early in the implementation of the study, at the first batch of intervention health centers, Kilo, Kalajärvi and Iso Omena, model elements which prioritize continuity of care had not yet been implemented. In subsequent interventions, this shortcoming was corrected and the elements of continuity of care were strengthened.

It may indeed be possible that both access and continuity of care were lowered due to the (external) influence of the chronic stages of the COVID-19 pandemic. Subsequently, however, we observed improvement to continuity of care at three intervention health centers, while remaining consistently low at all control health centers.

### Strengths

Intervention has been described in detail and documented on the degree of adherence to the main intervention. COC index was extracted from the electronic health record which increases its credibility. Value of main outcome measure T3 at baseline was similar in all health centers. Systems-related confounding factors were documented and described with detail. Intervention was conducted in a realistic environment and did not exclude any patient groups; this enables larger and easier scaling.

### Weaknesses

The centers in this study were not randomized but the populations however share similar baseline demographic and socio-economic factors. The value of main outcome measure COC at baseline was significantly better in Kilo and Kalajärvi health centers creating a bias flattering the intervention health centers’ results. T3 was calculated by hand by the medical officers; variability in the calculation methods is possible. T3 is also a problematic metric. In open access models it doesn’t consider the increased multidisciplinary consultations and decreased triage leading to the treatment of urgent and non-urgent needs within the same day. Thus, comparing T3 with usual appointment-based models (control health centers) creates a bias flattering the intervention health centers’ results. Unfortunately, we do not have a similar measure available that would calculate the availability of all appointment times (non-urgent and urgent) of control health centers. The fidelity of the intervention didn’t hold fully because of the realistic setting of the study. There was a need to further develop the intervention elements during the 1,5-year roll-out period to improve continuity of care and personnel well-being. The changes involved a shift from prioritizing rapid issue resolution during initial contact to emphasizing continuity of care by referring to the patient’s primary physician, with continuity becoming more valued over time. Also, at the start of the intervention roll-out, backlogs were addressed using additional resources. However, as the intervention elements became more familiar to the executive officers, the backlogs were managed by the health centers’ own personnel, and queues remained absent.

## Conclusions

Our research proposes the possibility of refining the primary care service model to yield better access without decreasing continuity. The main elements to improve access seemed to be same day scheduling, utilizing physicians in first contact to plan the patient’s journey, and aspiring to reach resolution upon the first contact. The intervention initially lacked an emphasis on continuity of care, which needs to be prioritized. Further research is needed to specify the components of team-based organization which enhance both access and continuity simultaneously.

## Supplementary Material

Appendix 2 Survey of Espoo health centres on the end of 2023 operating model for the team model study en.pdf

Appendix 1 A presentation of how tasks are approached in the former and new model of operation.pdf

## References

[1] STM. Tulevaisuuden sosiaali- ja terveyskeskus ohjelma; 2020. Available from: https://stm.fi/en/project?tunnus=STM012:00/2020.

[2] Tynkkynen LK, Pulkki J, Tervonen-Gonçalves L, et al. Health system reforms and the needs of the ageing population – an analysis of recent policy paths and reform trends in Finland and Sweden. Eur J Ageing. 2022;19(2):221–232. doi:10.1007/s10433-022-00699-x. 35465210 PMC9012246

[3] Keskimaki I, Tynkkynen L-K, Reissell E, et al. Finland: health system review. Health Syst Transit. 2019; Aug21(2):1–166. 31596240

[4] Saunes IS, Karanikolos M, Sagan A. Norway: health system review. Health Syst Transit. 2020;22(1):1–163. 32863241

[5] Satylganova A. Integrated care models: an overview. Copenhagen: WHO Regional Office for Europe; 2016.

[6] Levesque JF, Harris MF, Russell G. Patient-centred access to health care: conceptualising access at the interface of health systems and populations. Int J Equity Health. 2013;12(1):18. doi:10.1186/1475-9276-12-18. 23496984 PMC3610159

[7] Saultz JW. Defining and measuring interpersonal continuity of care. Ann Fam Med. 2003;1(3):134–143. doi:10.1370/afm.23. 15043374 PMC1466595

[8] Haggerty JL, Reid RJ, Freeman GK, et al. Continuity of care: a multidisciplinary review. BMJ. 2003;327(7425):1219–1221. doi:10.1136/bmj.327.7425.1219. 14630762 PMC274066

[9] Sandvik H, Hetlevik Ø, Blinkenberg J, et al. Continuity in general practice as predictor of mortality, acute hospitalisation, and use of out-of-hours care: a registry-based observational study in Norway. Br J Gen Pract. 2022;72(715):e84–90–e90. doi:10.3399/BJGP.2021.0340. 34607797 PMC8510690

[10] AFHTO. Optimizing the value of team-based primary care. Review of the literature [Internet]. Association of family health teams in; 2015. Ontario, Canada;. Available from: http://www.afhto.ca/wp-content/uploads/Optimizing-the-value-of-team-based-primary-care-LIT-REVIEW.pdf.

[11] Zygmunt A, Asada Y, Burge F. Is team-based primary care associated with less access problems and self-reported unmet need in Canada? Int J Health Serv. 2017;47(4):725–751. doi:10.1177/0020731415595547. 26182942

[12] Wensing M, Szecsenyi J, Stock C, et al. Evaluation of a program to strengthen general practice care for patients with chronic disease in Germany. BMC Health Serv Res. 2017;17(1):62. doi:10.1186/s12913-017-2000-2. 28109281 PMC5251235

[13] Baxter S, Johnson M, Chambers D, et al. The effects of integrated care: a systematic review of UK and international evidence. BMC Health Serv Res. 2018;18(1):350. doi:10.1186/s12913-018-3161-3. 29747651 PMC5946491

[14] Szafran O, Kennett SL, Bell NR, et al. Patients’ perceptions of team-based care in family practice: access, benefits and team roles. J Prim Health Care. 2018;10(3):248–257. doi:10.1071/HC18018. 31039939

[15] Forman JH, Robinson CH, Krein SL. Striving toward team-based continuity: provision of same-day access and continuity in academic primary care clinics. BMC Health Serv Res. 2019;19(1):2. doi:10.1186/s12913-019-3943-2. 30832649 PMC6399842

[16] Arnetz BB, Goetz CM, Arnetz JE, et al. Enhancing healthcare efficiency to achieve the Quadruple Aim: an exploratory study. BMC Res Notes. 2020;13(1):362. doi:10.1186/s13104-020-05199-8. 32736639 PMC7393915

[17] Sikka R, Morath JM, Leape L. The Quadruple Aim: care, health, cost and meaning in work. BMJ Qual Saf. 2015;24(10):608–610. doi:10.1136/bmjqs-2015-004160. 26038586

[18] Wagner EH, Austin BT, Von Korff M. Organizing care for patients with chronic illness. Milbank Q. 1996;74(4):511–544. doi:10.2307/3350391. 8941260

[19] Murray M, Berwick DM. Advanced Access: reducing waiting and delays in primary care. JAMA. 2003;289(8):1035–1040. doi:10.1001/jama.289.8.1035. 12597760

[20] Ansell D, Crispo JAG, Simard B, et al. Interventions to reduce wait times for primary care appointments: a systematic review. BMC Health Serv Res. 2017;17(1):295. doi:10.1186/s12913-017-2219-y. 28427444 PMC5397774

[21] Winner LE, Reinhardt E, Benishek L, et al. Lean management systems in health care: a review of the literature. Qual Manag Health Care. 2022;31(4):221–230. doi:10.1097/QMH.0000000000000353. 35180733

[22] THL. Finnish Institute for Health and Welfare. Access to treatment in primary health care. Finnish Institute for Health and Welfare (in Finnish) [Internet]. Available from: https://thl.fi/tilastot-ja-data/tilastot-aiheittain/terveyspalvelut/hoitoonpaasy-perusterveydenhuollossa.

[23] Marttila T, Mahkonen R, Pyrhönen K. Hoitoonpääsy perusterveydenhuollossa 2024; 2024. THL. Available from: https://urn.fi/URN:NBN:fi-fe2024060343152.

[24] Ministry of Social Affairs and Health. Health Care Act 1326/2010 English; 2010.

[25] Moore GF, Audrey S, Barker M, et al. Process evaluation of complex interventions: medical Research Council guidance. BMJ. 2015;350(6):h1258–h1258. doi:10.1136/bmj.h1258. 25791983 PMC4366184

[26] Van Walraven C, Oake N, Jennings A, et al. The association between continuity of care and outcomes: a systematic and critical review. J Eval Clin Pract. 2010;16(5):947–956. doi:10.1111/j.1365-2753.2009.01235.x. 20553366

[27] Bice TW, Boxerman SB. A quantitative measure of continuity of care. Med Care. 1977;15(4):347–349. doi:10.1097/00005650-197704000-00010. 859364

[28] Malmivaara A. Assessing validity of observational intervention studies – the Benchmarking Controlled Trials. Ann Med. 2016;48(6):440–443. doi:10.1080/07853890.2016.1186830. 27238631 PMC5152539

[29] Kortelainen M, Salokangas H. Kvasikokeelliset menetelmät terveydenhuollon ja terveystaloustieteen vaikutusarvioinneissa. Sos Aikakauslehti. 2023;60(3):29. https://journal.fi/sla/article/view/122529

[30] Sosiaali- ja terveysministeriö. Hoitoon pääsy (hoitotakuu) [Internet]. Sosiaali- ja terveysministeriö; Available from: https://stm.fi/hoitotakuu.

[31] Wu FM, Slightam CA, Wong AC, et al. Intensive outpatient program effects on high-need patients’ access, continuity, coordination, and engagement. Med Care. 2018;56(1):19–24. doi:10.1097/MLR.0000000000000833. 29087980

[32] Davie S, Kiran T. Partnering with patients to improve access to primary care. BMJ Open Qual. 2020;9(2):e000777. doi:10.1136/bmjoq-2019-000777. PMC7170539 32241765

[33] Cook LL, Golonka RP, Cook CM, et al. Association between continuity and access in primary care: a retrospective cohort study. CMAJ Open. 2020;8(4):E722–E730. doi:10.9778/cmajo.20200014. PMC7676991 33199505

[34] Lautamatti E, Mattila K, Suominen S, et al. A named GP increases self-reported access to health care services. BMC Health Serv Res. 2022;22(1):1262. doi:10.1186/s12913-022-08660-5. 36261827 PMC9580200

[35] Tuompo W, Timonen M, Ruotsalainen K, et al. Väestön jakaminen omalääkäreille parantaa hoidon jatkuvuutta. Finn Med J; [cited 2025 Jan 22]. Available from:www.laakarilehti.fi/e42796.

[36] Michael M, Schaffer SD, Egan PL, et al. Improving wait times and patient satisfaction in primary care. J Healthc Qual. 2013;35(2):50–60. doi:10.1111/jhq.12004. 23480405

[37] Gulliford MC. Availability of primary care doctors and population health in England: is there an association? J Public Health Med. 2002;24(4):252–254. doi:10.1093/pubmed/24.4.252. 12546200

[38] Chan KS, Wan EYF, Chin WY, et al. Association between team-based continuity of care and risk of cardiovascular diseases among patients with diabetes: a retrospective cohort study. Diabetes Care. 2022;45(5):1162–1169. doi:10.2337/dc21-1217. 35263428

[39] Germack HD, Leung L, Zhao X, et al. Association of team-based care and continuity of care with hospitalizations for veterans with comorbid mental and physical health conditions. J Gen Intern Med. 2022;37(1):40–48. doi:10.1007/s11606-021-06884-5. 34027614 PMC8739416

[40] Xu W, Yu EYT, Chin WY, et al. Team-based continuity of care for patients with hypertension: a retrospective primary care cohort study in Hong Kong. Br J Gen Pract. 2023;73(736):e807–15–e815. doi:10.3399/BJGP.2023.0150. 37845086 PMC10587903

