# Supplementary material for: Improving access, mixed continuity: effects of multidisciplinary teams on primary health-care in Finland – a quasi-experimental study
Source: Scand J Prim Health Care. 2025 May 8;43(4):745–58. doi: 10.1080/02813432.2025.2502658 (PMC12632202; doi:10.1080/02813432.2025.2502658)
Supplement: Appendix 2 Survey of Espoo health centres on the end of 2023 operating model for the team model study en.pdf [file IPRI_A_2502658_SM4757.pdf]

Survey for Espoo health centres in 2023 from the team model study

The questionnaire is completed jointly by the station's multidisciplinary management team. For each item, please select the description that **best** describes **your activities** and the way your health centre will operate at the end of 2023, even if not in all respects. If you wish, you can elaborate on the answer to each question in the Further information section below.

Responses will be handled as part of the team model survey and only by the research team and will not be shared with anyone outside the survey.

Research permit: LUVN slide number 67/13.01.00/2024 Research team,

Elisa Jokelin (Principal Investigator, for more information please contactelisa.jokelin@helsinki.fi , tel. 040 7380 301)
Laura Piirainen
Erja Mustonen
Paulus Torkki

* Mandatory

1. Health centre to which the answers apply *

2. Multidisciplinarity and roles in resolving a client's case *

- ◯ Immediate assistance from the most appropriate professional in the team
- ◯ A team of different professionals, managing customer pathways
- ◯ Multidisciplinary team at the heart of the operation, availability maintained at all times, immediate resolution of the issue, management of disruptive demand
- ◯ Consultation channels, consultant available
- ◯ Management in professional groups, urgency classification in place, issues resolved at the office, nurse on call, smooth operation of the emergency service

3. More information on Multidisciplinarity and roles in resolving a client's case

4. Continuity of care *

- ◯ Continuity of care is maintained at every contact with all professionals
- ◯ GPs and nurses appointed, maintained mainly for long-term conditions
- ◯ Continuity is monitored and managed, team has indicators for continuity, processes are taken into account
- ◯ Continuity is known, only occasional, not managed
- ◯ Continuity is not monitored or managed

5. Further information under Continuity of care

6. Development *

- ◯ Professionals work with the client to develop a proactive and preventive approach. The thing or service the client needs is provided when they need it. Development aims at perfection
- ◯ Every day, as part of everyday life, at the customer interface, based on knowledge, developed towards strategic objectives. Means of streamlining operations widely managed. Customers get things done without bottlenecks, unnecessary processes removed from
- ◯ Sessions disconnected from everyday life. Deliberately eliminate waste and identify unnecessary parts of the customer's path
- ◯ Projects and/or management's role. Individual processes streamlined
- ◯ No or random, often not related to strategy. There is some know-how on streamlining but it is not exploited

7. More information under Development

8. Management *

- ◯ Information is used to predict the future, which is managed proactively, with leaders setting the direction
- ◯ Strategic objectives decomposed to process level, bottom-up development and information flow, daily management, automated data. Professionals are coached towards strategic objectives
- ◯ Core objectives broken down to different levels, strategy understandable, time taken to collect data. Managers ask questions and help professionals to solve problems themselves
- ◯ Separate objectives and day-to-day activities. Leaders lead professional teams, spend time solving and developing themselves
- ◯ No targets, lots of data but little/no meaningful analysed data, management based on gut feeling, middle management no visibility of the situation in the unit. Leaders' time is wasted on fire extinguishing and there is a lot of overtime

9. More information under: Governance

10. Supply and demand *

- ◯ Meeting clients' needs before they contact us, finding those in need of care
- ◯ Multidisciplinary first contact. Care for the long-term sick planned and care pathways built to support self-care and health promotion
- ◯ Demand is met in real time, so there are no queues. No rejection
- ◯ Remote consultations, consultant in place, no multidisciplinarity in first contact otherwise
- ◯ Queues are created

11. More information on Demand and supply

This is not content created or recommended by Microsoft. The information you submit will be sent to the form owner.

Microsoft Forms
