# Supplementary material for: Improving access, mixed continuity: effects of multidisciplinary teams on primary health-care in Finland – a quasi-experimental study
Source: Scand J Prim Health Care. 2025 May 8;43(4):745–58. doi: 10.1080/02813432.2025.2502658 (PMC12632202; doi:10.1080/02813432.2025.2502658)
Supplement: Appendix 1 A presentation of how tasks are approached in the former and new model of operation.pdf [file IPRI_A_2502658_SM4756.pdf]

Appendix 1: A presentation of how tasks are approached in the former and new model of operation.

Task	Former mode of operation, control health centers	New mode of operation, intervention health centers
Solution to service need	The nurse evaluates the need for treatment from a medical perspective. The reception appointment is evaluated by the urgency classification (acute/non-urgent).	The team starts responding to the service need as soon as the patient makes contact. The perspective of assessing the need for treatment changes and a right professional and right tool is found to answer the patient's need/concern. Part of the solution can be, for example, on-site or remote reception or consultation. The matter is postponed to the future if additional information is needed to support decision-making (e.g. diagnostics), the client needs to be examined at an in-person reception, which the patient cannot access today, or to ensure the continuity of care, a certain professional who is not available now is wanted to be used.
Remote care	If the matter cannot be resolved by the nurse during the first contact, the patient is generally reserved an in-person appointment with a nurse or doctor.	The patient is asked to attend the reception in person if the medical treatment requires it (need for a clinical examination or communication problems). Proactive monitoring is emphasized in remote care. It depends on the patient, the matter and the professional's experience, how much can be treated remotely. Remote care is monitored with a meter. The operation has become more remote and consultation-oriented.
Responding to demand	The aim is to handle all patient calls during the same day. It is often not possible, so calls from the non-urgent line remain in the queue. The need for a reception is sought to be ensured only for urgent matters or matters that require immediate treatment. Other demand is directed to the future, forming queues.	The aim is to avoid queuing and to get things done as far as possible in the first contact. Visual day-to-day management, metrics, continuous deviation collection and problem solving support this. The care of all patients is either started or completed during the same day without any urgency rating. The immediate and urgent need for care is secured, so calls from the non-urgent line can remain in the queue. If it is not possible to meet the entire demand, problem solving is started, the reasons are analyzed and possible corrective measures are evaluated.
Ensuring continuity of care	No named responsible nurses or doctors. Monitoring of type 2 diabetics is centralized to public health nurses. By chance, nurses familiar with long-term illnesses (e.g. asthma). A responsible doctor has not been named, but in non-urgent matters, an appointment with a familiar doctor is prefered, if appointments are available.	All patients are assigned an own responsible nurse as the contact person. According to needs determined by the patient's long-term illness and the personnel structure of the health center, a nurse, a specially trained nurse or a public health nurse is determined to be the responsible nurse. Calls are routed to the nurse's line and, if necessary, another nurse from the same team answers the call. As a rule, proactive remote monitoring is reserved to same responsible professional.
Multiprofessional teamwork	Each professional works in own room, where receptions are also conducted. Consultation is carried out through various channels: door-to-door consultation, phone, Teams, message or reservation in the patient information system. At some health centers, more consultations are carried out, at others more in-person appointments for the same or a different professional are reserved. The patient coordinates his/her own treatment.	Nurses and supervising doctor/doctors work in the same team room. Nurses can consult the supervising doctor in the team room or leave a message to the doctor who has handled the client's case before. Reception rooms are for receptions and quiet working. The responsible nurse acts as the coordinator of the treatment.
Development	Development happens on a weekly or monthly basis, mainly in professional group meetings or with different projects. The development targets are randomly selected.	Operations are developed daily and constantly improved. Problem solving is systematic. Development starts from strategic goals, deviations or improving core processes.
Management	Goals, their operationalization into daily goals and the metrics that describe them are not defined. Monitoring of a single meter (e.g. T3).	Visual day-to-day management and daily meetings in the team. The team monitors process indicators on a daily basis, and the management of the health center monitors strategic performance metrics. The supervisors and middle management of the units evaluate the service level performance on a daily basis.
